# Safe transanal drainage tube placement using a tapered-tip sheath system for obstructive colorectal cancer

**DOI:** 10.1055/a-2587-9144

**Published:** 2025-05-14

**Authors:** Fumioki Toyoda, Masataka Yokode, Tomoaki Matsumori, Munehiro Ikeda, Takahiro Utsumi, Yuki Nakanishi, Hiroshi Seno

**Affiliations:** 1 Department of Gastroenterology and Hepatology, Kyoto University Graduate School of Medicine, Kyoto, Japan

A 67-year-old woman presented at our hospital with abdominal bloating and pain. Abdominal computed tomography revealed sigmoid colon thickening and proximal bowel dilatation ( Fig. 1 ). Emergency colonoscopy revealed advanced colorectal cancer with almost complete obstruction ( Fig. 2 ). Consequently, endoscopic decompression using a transanal drainage tube (TDT) (Argyle Fukuroi Dennis Colorectal tube, Cardinal Health) was attempted ( Fig. 3 ). However, the equipped 0.055-inch guidewire could not pass through the stenosis because of its stiffness. Therefore, a 0.035-inch hydrophilic biliary guidewire (Hydra Jagwire, Boston Scientific) preloaded through a biliary catheter (MTW, MTW Endoskopie) was used and successfully passed through the stenosis. The biliary catheter was then exchanged with a tapered-tip sheath system (EndoSheather, Piolax) composed of a tapered inner catheter and outer sheath ( Fig. 4 **a, b** ). The system passed smoothly through the stenosis. After the inner catheter and biliary guidewire were withdrawn, the equipped guidewire was inserted into the outer sheath ( Fig. 4 **c** , Fig. 5 **a, b** ). Subsequently, the colonoscope and outer sheath were removed, and the sigmoid colon was straightened using the equipped guidewire. Finally, the TDT was successfully placed ( Fig. 5 **c** , Video 1 ).

**Fig. 1:**
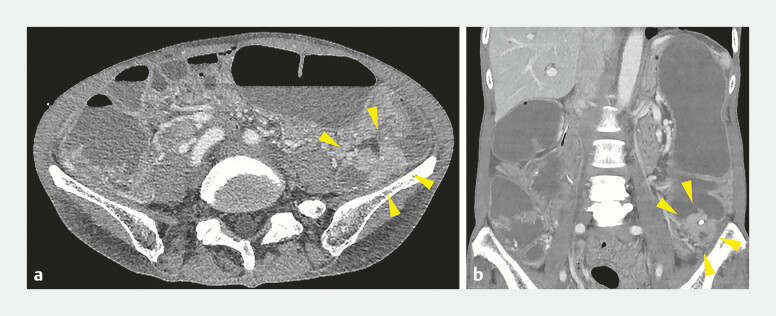
Abdominal contrast-enhanced computed tomography images. Horizontal ( **a** ) and coronal ( **b** ) images show wall thickening in the sigmoid colon (yellow arrowheads) and proximal bowel dilatation.

**Fig. 2:**
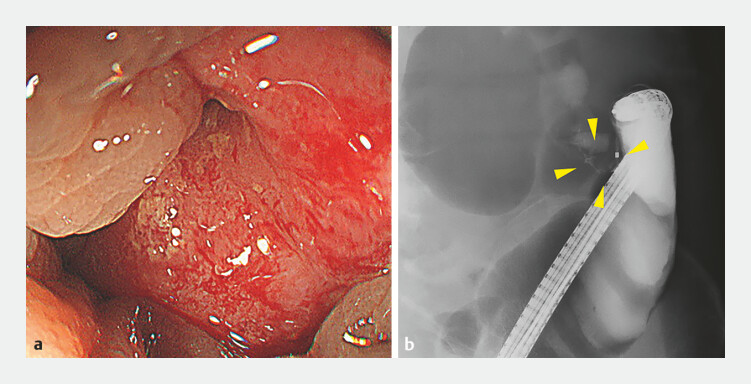
Colonoscopy and fluoroscopy. **a** Colonoscopy image showing almost complete bowel obstruction caused by colorectal cancer. **b** Fluoroscopic image of severe stenosis due to the tumor (yellow arrowheads).

**Fig. 3:**
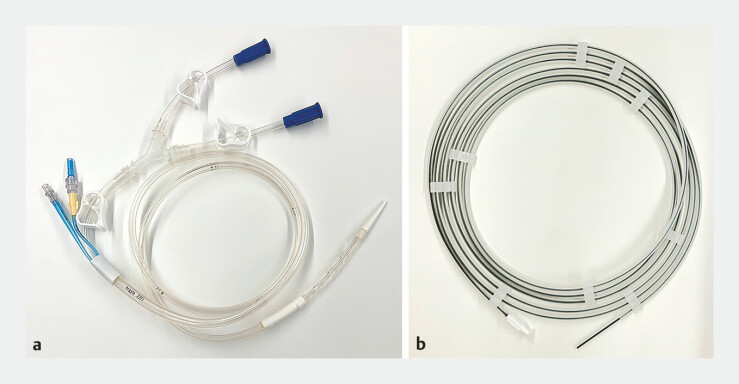
Images of the transanal drainage tube. **a** Overall view of a transanal drainage tube (Argyle Fukuroi Dennis Colorectal Tube, Cardinal Health). **b** The 0.055-inch guidewire equipped with the tube.

**Fig. 4:**
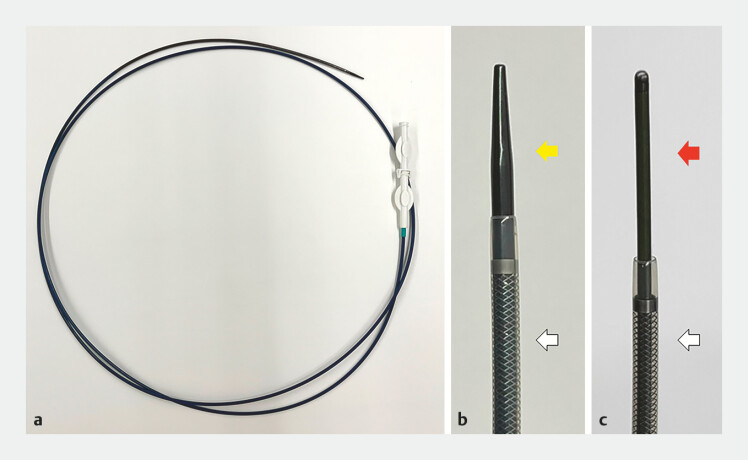
Images of the tapered-tip sheath system. **a** )Overview of the tapered-tip sheath system (EndoSheather, Piolax). **b** The tapered-tip inner catheter tip (yellow arrow) and outer sheath (white arrow). ( **c** ) Tip of the 0.055-inch equipped guidewire (red arrow) and outer sheath (white arrow).

**Fig. 5:**
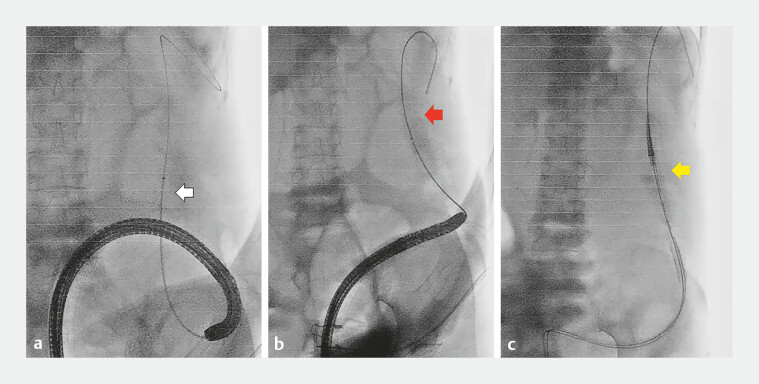
Fluoroscopic images of transanal drainage tube placement. **a** Placement of the tapered-tip sheath system over the hydrophilic biliary guidewire (white arrow). **b** Insertion of the 0.055-inch guidewire (red arrow) into the outer sheath. **c** Insertion of the transanal drainage tube (yellow arrow) through the stenosis over the 0.055-inch guidewire.

This video shows the procedure for transanal drainage tube placement using a tapered-tip sheath system for obstructive colorectal cancer.Video 1

TDT placement is effective in preventing emergency surgery for patients with malignant colorectal obstruction 1 2 . The equipped guidewire is rigid enough to facilitate TDT placement; however, its stiffness may make it difficult to pass through the stenosis and may cause perforation 3 . Hydrophilic biliary guidewires are useful in such cases 4 , but cannot be exchanged with an equipped guidewire through a biliary catheter because of their thickness. As in this case, the tapered-tip sheath system, first developed for bile duct biopsy 5 , enables a hard-equipped guidewire to pass through the malignant colorectal stenosis. This system may be useful for safe TDT placement in cases of unsuccessful insertion of an equipped guidewire using the usual method.

Endoscopy_UCTN_Code_TTT_1AQ_2AF

## Notes

Endoscopy E-Videos https://eref.thieme.de/e-videos
*E-Videos* is an open access online section of the journal *Endoscopy* , reporting on interesting cases and new techniques in gastroenterological endoscopy. All papers include a high-quality video and are published with a Creative Commons CC-BY license. Endoscopy E-Videos qualify for HINARI discounts and waivers and eligibility is automatically checked during the submission process. We grant 100% waivers to articles whose corresponding authors are based in Group A countries and 50% waivers to those who are based in Group B countries as classified by Research4Life (see: https://www.research4life.org/access/eligibility/ ). This section has its own submission website at https://mc.manuscriptcentral.com/e-videos .
